# Choice of Biologic Therapy for Patients with Rheumatoid Arthritis: The Infection Perspective

**DOI:** 10.2174/157339711794474620

**Published:** 2011-02

**Authors:** Filip De Keyser

**Affiliations:** Department of Rheumatology, Ghent University, Belgium

**Keywords:** Rheumatoid arthritis, infection, biologicals, targeted therapies, TNF inhibitors.

## Abstract

Biologicals revolutionized the treatment of Rheumatoid Arthritis (RA). The targeted suppression of key inflammatory pathways involved in joint inflammation and destruction allows better disease control, which, however, comes at the price of an elevated infection risk due to relative immunosuppression. The disease-related infection risk and the infection risk associated with the use of TNF-α inhibitors (infliximab, adalimumab, etanercept, golimumab and certolizumab pegol), rituximab, abatacept and tocilizumab are discussed. Risk factors clinicians need to take into account when selecting the most appropriate biologic therapy for RA patients, as well as precautions and screening concerning a number of specific infections, such as tuberculosis, intracellular bacterial infections, reactivation of chronic viral infections and HIV are reviewed.

## INTRODUCTION

The introduction of biological therapies targeting specific inflammatory mediators revolutionized the treatment of rheumatoid arthritis (RA). Targeting key components of the immune system allows efficient suppression of the pathologic inflammation cascade that gives rise to RA symptoms and subsequent joint destruction. As flip side of the coin, treatment with biologicals leaves the patient more susceptible to infection by inducing a certain extent of immuno-suppression. 

The expanding compendium of targeted therapies for RA includes inhibitors of TNF-α (infliximab, adalimumab, etanercept and the newer antibodies golimumab and certolizumab pegol), rituximab which targets the B-cell specific CD 20 antigen, the T cell costimulation inhibitor abatacept and the IL-6 receptor inhibitor tocilizumab. Although much remains to be discovered about the precise mechanisms of increased infection risk under biologic therapy, it is clear that clinical differences with respect to type and frequency of infectious complications exist between the different compounds.

This article aims to summarize literature data on compound-related and disease-related infection risk factors that clinicians need to take into account when selecting the most appropriate biologic therapy for their RA patients. The risk of serious infections associated with different biologicals is discussed, followed by risks and precautions needed under biological therapy with respect to a number of specific infections, such as tuberculosis, intracellular bacterial infections, reactivation of chronic viral infections and HIV.

## DISEASE-RELATED RISK OF INFECTION

RA is known to be associated with an increased risk of infection [1,2], although it is difficult to distinguish the infection risk associated with the disease per se from the therapy-associated infection risk. Older studies suggest that RA intrinsically entails an elevated susceptibility to infection [3], probably through RA-associated changes in the cellular immune response [2]. A large population-based retrospective study comparing RA patients with matched controls reported a nearly doubled incidence of documented infections in RA patients [1]. RA severity indices, such as presence of rheumatoid factor, increased sedimentation rate and extra-articular involvement are predictors of serious infection episodes in RA, in addition to corticosteroid use and the presence of comorbidities [4]. Infection is also partly responsible for the excess mortality rate in RA patients, with infection-related standardised mortality rates in RA patients ranging from 4.2 to 14.9 [5]. 

## THERAPY-RELATED RISK OF INFECTION

### General Appraisal of Serious Infection Risk

The infection risk associated with RA treatment should always be evaluated against the background of the intrinsically increased baseline risk of infection in RA patients.

Corticosteroids, some disease-modifying antirheumatic drugs (DMARDs) and targeted biologic therapies all have a negative impact on the capacity of RA patients to mount an adequate immune response and therefore superimpose additional infection risk to the intrinsically increased infection susceptibility of this patient population. 

Corticosteroids are well-known to increase infection risk by inducing immunosuppression. The degree to which they suppress immune competence increases with the dose and duration of treatment. Treatment for longer than 2 weeks with over 20 mg/day of prednisolone or equivalent is commonly considered to induce clinically significant immuno-suppression [6], whereas a meta-analysis showed that cumulative doses below 500 mg or mean daily doses below 10 mg do not increase the risk of infectious complications and can be considered as not immunosuppressive [7]. 

Corticosteroids and combination therapy of corticosteroids and conventional DMARDs were shown to increase the risk of serious infections in RA patients, but non-biological DMARD therapy without corticosteroids was not associated with increased incidence of infection [4,8], although some DMARDS (methotrexate, azathioprine, leflunomide, cyclophosphamide, cyclosporine) have well-known negative effects on the immune system. Hydroxychloroquine, sulfasalazine, and gold salts do not have immunosuppressive effects.

Biological therapies specifically inhibiting targeted molecules of the immune system allow far better disease control, at the expense of an increased risk of infections (reviewed in [9]).

Most of the available data on the infection risk of targeted therapies concern inhibitors of tumor necrosis factor alpha (TNF-α), which have been in clinical use the longest, while information on the newer biologicals is much more limited. Infectious complications of biological therapy include bacterial infections, such as tuberculosis, Streptococcus pneumoniae and Listeria monocytogenes and potential reactivation of viral infections such as hepatitis B or C, herpes and varicella zoster.

### TNF Inhibitors

TNF-α is a cytokine secreted by macrophages in response to inflammatory stimuli and is involved in immune regulation and inflammation as well as in sepsis, apoptotic cell death and cancer. TNF inhibitors were the first class of biological agents on the market for the treatment of RA, with the first agent etanercept introduced in 1998, so we can now look back on a decade of clinical experience with these products. Most of the data available concern the first three products of this therapeutic class: etanercept, a recombinant soluble decoy TNF-receptor; infliximab, a chimeric monoclonal anti-TNF antibody; and adalimumab, a fully human anti-TNF monoclonal antibody. Studies directly comparing the different TNF-inhibitors are lacking, but a recent network meta-analysis covering Cochrane reviews on different biologicals for RA found a reduced therapy withdrawal rate for adverse events under etanercept as compared with infliximab and adalimumab [10].

Although the incidence of infections and serious infections (defined as life-threatening, requiring hospitalization or intravenous antibiotics) in the randomized controlled registration trials of the first 3 TNF inhibitors etanercept, infliximab and adalimumab mostly did not report significant increases in infection risk with these products in comparison with controls [9], epidemiological studies as well as registry data have revealed increased incidences of infection with these compounds (reviewed in [9]). 

A meta-analysis of serious infections in 9 randomized controlled trials with the anti-TNF antibodies infliximab and adalimumab found an odds ratio of serious infections of 2.01 (95% CI 1.31-3.09) for patients treated with anti-TNF anti-bodies for at least 12 weeks, in comparison with a control population treated with placebo or placebo in combination with DMARDs [11]. These findings contrast with a more recent and broader (including etanercept) meta-analysis by Leombruno *et al*., who report only non-significant increases in serious adverse event and infection rates under anti-TNF therapy [12]. These discrepancies may be explained by different study inclusion criteria, but also by the inclusion of more recent trials, where increased awareness of possible infectious complications with anti-TNF therapy led to more stringent patient screening and selection.

In the German RABBIT registry study the relative risks for serious infection was 2.82 (95% CI 1.4–5.9) in etanercept-treated patients (corresponding to 15.73 [95% CI 12.6–19.7] episodes/100 patient-years) and 2.70 (95% CI 1.3–5.9) under infliximab treatment (corresponding to 20.59 [95% CI 16.2–26.2] episodes/100 patient-years) in comparison with patients treated with conventional DMARDs, where the incidence rate of serious infections was 5.08 (95% CI 3.5–7.3) per 100 patient-years [13]. In the British Society for Rheumatology Biologics Register, overall serious infection rates during anti-TNF therapy compared with DMARD treatment were not increased (IRR 1.03, 95% CI 0.68–1.57), but in contrast anti-TNF therapy increased the rate of serious skin and soft tissue infections (IRR of 4.28, 95% CI 1.06–17.17) [14]. A Swedish observational study reported an increased relative risk for hospitalisation due to infection during the first year of anti-TNF treatment, which subsided with increasing duration of treatment [15]. 

A systematic retrospective analysis in a tertiary clinical center revealed an increased incidence of serious infections during the first course of anti TNF-therapy (10.5 +/- 86.9 per 100 patient-years, in comparison with 3.4 +/- 38.7 before TNF-therapy), with a number needed to harm of 14 [16]. A recent Italian registry study reported an incidence rate of 3.59 serious infections per 100 patient-years (95% CI 2.77-4.41) in the first 36 months of anti-TNF therapy, without significant differences in incidence and type of infection between the different anti-TNF agents [17].

A recent study using data from the North American CORRONA registry indicates that MTX and TNF inhibitor therapy and the combination of both are all associated with a comparable increase in the incidence of overall infections as well as opportunistic infections [18]. 

Data on the infectious complication risk with the newer TNF-inhibitors golimumab and certolizumab are still limited. Golimumab is a fully human anti-TNF monoclonal anti-body, whereas certolizumab pegol consists of a humanized Fab-fragment fused to polyethylene glycol (PEG) moiety. Replacement of the Fc-fragment by PEG may avoid Fc-mediated side effects such as complement activation, may contribute to its preferential distribution to inflamed tissues and increases the half-life of certolizumab pegol to 14 days.

RCTs with golimumab (reviewed in [19]) report serious infection rates of 0.98 to 2.44 percent over 24 weeks [20-22], with one study observing serious infections in 2.19% of patients over a one-year period [23]. These figures are in range with what has been reported for other anti-TNF agents. In the FAST4WARD study, monotherapy with certolizumab pegol yielded a serious infection incidence rate of 1.8%, whereas combination of certolizumab with MTX induced serious infections in 2.85 and 4.99% of treated patients [24,25].

### Rituximab

Rituximab is a genetically engineered chimeric monoclonal antibody that targets CD20-positive B cells. By binding to CD20, rituximab depletes subpopulations of peripheral B cells through different mechanisms, including cell-mediated and complement-dependent cytotoxicity and promotion of apoptosis. B cells can contribute to the initiation and maintenance of the inflammatory cascade in RA by acting on antigen presentation by T cells and through production of pro-inflammatory cytokines and auto-antibodies.

The incidence of serious infections under rituximab treatment appears to be rather limited: 1.27 to 2.27% over 24 weeks [26,27], 4.96% over 48 weeks [28]. A recent meta-analysis reported that the overall pooled odds ratio for serious infection under rituximab treatment was not significantly increased (OR 1.45, 95% CI 0.56-3.73) [29]. All serious infections occurred in patients treated with the highest (2 times 1000 mg) dose of rituximab [29]. Although the overall increase in infection risk under rituximab seems to be limited, rituximab treatment has been associated with rare cases of progressive multifocal leukoencephalopathy** (**PML) (read further).

### Abatacept

The T cell costimulation modulator abatacept is a fully human soluble fusion protein that consists of the extra-cellular domain of human CTLA-4 linked to the modified Fc portion of human IgG1. Upon antigen recognition T cells require a costimulatory signal for full activation. Like the natural CTLA4 molecule, abatacept interferes with the CD80/CD86 binding to T cell CD28 with higher avidity than CD28.

The limited data available on abatacept suggest that the risk of serious infections with these products may be more limited than that of the TNF inhibitors. Abatacept phase III RCT’s reported serious infection incidences of 2.33% [30] and 2.39% [31] over 26 weeks, and 2.54% [32] to 3.13% [33] over one year. A five year extension of a 1 year double blind RCT reported 3.0 serious infections per 100 patient-years over the whole study period, *versus* 2.1/100 patient-years in the first year of the study [34]. In the ATTEST trial which compared the efficacy and safety of infliximab and abatacept plus MTX in patients with insufficient response to MTX alone, considerably lower rates of serious infections were observed under abatacept treatment (1.9 *versus* 8.5%) [35] A recent meta-analysis by Salliot *et al*. found that abatacept did not significantly increase the risk of serious infections in RA patients [29].

The incidence of serious infection episodes does not increase with prolonged abatacept treatment, as evidenced by the open label extension studies of the AIM trial, reporting 4.3 [36] and 3.0 [34] serious infections per 100 patient-years after 2 and 5 years of treatment, respectively, in comparison with 4.2 serious infections per 100 patient-years observed in the 1 year double blind phase of the study [37,38].

Combination of abatacept with etanercept yielded little clinical benefit, but did increase the incidence of serious infections (3.5% in the combination group versus 0% in the etanercept group) [39]. This study confirmed earlier findings that abatacept in combination with another biological agent increased the incidence of serious adverse events, including serious infections [33].

### Tocilizumab

Tocilizumab is a humanised monoclonal antibody targeting the interleukin-6 receptor, which can be found both on cell surfaces and in the circulation. Tocilizumab blocks the downstream effects of IL-6, a cytokine with pleiotropic effects that contributes to the inflammation cascade in RA, by affecting the function of neutrophils, T cells, B cells, monocytes, and osteoclasts. Additionally, IL-6 is a potent inducer of the hepatic acute phase response. 

The risk of serious infections under tocilizumab treatment reported in RCTs is relatively low, with figures reported ranging from 2.29 to 9.98 per 100 patient-years [40]. However, a number of these studies excluded patients with a history of infections or increased infection risk, so further evidence from clinical practice or registry studies is needed in order to assess the real-life infection risk associated with tocilizumab.

## SPECIFIC INFECTIONS UNDER BIOLOGICAL THERAPY

The most common sites of infections associated with biological therapy are respiratory tract infections - including pneumonia - septic arthritis, skin and soft tissue infections, and urinary tract infections [9]. As TNF plays an important role in the host defense mechanism against intracellular pathogens [41,42], anti-TNF therapy is associated with increased risk of infection with intracellular micro-organisms, such as Mycobacterium tuberculosis, Listeria monocytogenes and Legionella pneumophila. 

### Intracellular Bacterial Infections

Biological therapies for RA are associated with an increased risk of **tuberculosis**, mainly by reactivation of a latent Mycobacterium tuberculosis infection. The impact of biological therapies on tuberculosis risk must, however, be evaluated against the background of increased incidence of tuberculosis (TB) due to RA itself and regional differences in exposure to Mycobacterium tuberculosis [43-46]. Conventional DMARDs and corticosteroids are also associated with an increased risk of tuberculosis [47].

A Swedish study over the period 2000-2001 reported a 4-fold increase in TB risk for RA patients treated with TNF antagonists [48], whereas a Korean study observed a relative risk of TB of 8.9 for RA patients and 30.1 for RA patients treated with infliximab in comparison with the general population [49]. In the Spanish biologicals register BIOBADASER, annual TB incidence rates of 1893 and 1113 per 100 000 were reported in the year 2000 and 2001 respectively, in anti-TNF treated RA patients, in comparison with 95/100 000 in RA patients not treated with TNF inhibitors and 20/100 000 in the general population [50,51].

Reactivation of latent tuberculosis emerged as an adverse event from early clinical experience with the first generation TNF antagonists (reviewed in [52,53]), concurrent with the important role of TNF in the immune response to mycobacteria [54,55]: TNF stimulates phagocytosis of mycobacteria by macrophages and enhances mycobacterial killing in concert with IFN-γ, is crucial in recruitment of inflammatory cells and stimulates chemokine production [59,60]. TNF further plays a key role in confining mycobacteria to granulomas and achieving a latent state of the disease, which may explain both the timing of disease reactivation,usually observed within the first months of treatment, and the difference between the different TNF antagonist, which display different kinetics leading to different TNF bioavailability in granulomatous tissue [56,57].

Later trials with newer biologicals have used TB screening and prophylaxis or excluded patients with evidence of previous TB exposure and hence reported much lower TB incidence rates. The impact and importance of TB screening and prophylaxis is further illustrated by the drastic decrease in TB cases after implementation of TB screening and prophylaxis guidelines [50,58]. The majority of TB cases in anti-TNF treated patients afterwards were due to incorrect implementation of TB screening and prophylaxis guidelines [42,50]. TB risk with the anti-TNF antibodies infliximab and adalimumab is higher than with the fusion protein etanercept [42]. A recent study presenting long-term follow-up data on patients with TB as a complication of TNF blocker therapy shows that biological therapy can be safely resumed after adequate treatment of TB [41].

The tuberculosis risk associated with rituximab is currently unknown. No tuberculosis was reported in rituximab RCTs [26-28]. The consensus statement on the use of biologicals in RA warns against the use of rituximab in the presence of serious or opportunistic infections [59], but some case reports described the use of rituximab without adverse consequences in patients with a history of active TB [60,61]. 

The risk for TB reactivation associated with abatacept therapy currently remains unknown, but one case of tuberculosis has been observed in phase III trials with this drug [32]. Although the B7/CD28 T cell costimulation pathway plays a role in the granulomatous response to mycobacterium infection [62], abatacept did not exacerbate mycobacterium tuberculosis infection in mice, in contrast with anti-TNF treatment [63]. The clinical significance of these experimental findings remains to be investigated, however. Therefore, TB screening prior to abatacept therapy is recommended until the TB reactivation risk is known [59]. 

For the recently introduced IL-6 receptor antagonist tocilizumab the risk of tuberculosis reactivation appears to be low. No cases of tuberculosis reactivation under tocilizumab treatment were reported up to now [40,64-69], despite the fact that most clinical trials with tocilizumab did not perform tuberculosis screening or prophylaxis and tuberculosis was an exclusion criterion in only two trials [68,69]. In view of the well-established role of IFN-γ production in the antituberculosis immune response [70], *in vitro* findings that tocilizumab, in contrast with infliximab and etanercept, does not impair IFN-γ production in response to mycobacterial antigen exposure [71], and Mycobacterium tuberculosis-induced interleukin 6 inhibits the responsiveness of macrophages towards IFN-γ [72], may suggest a low risk for TB reactivation during tocilizumab therapy. The clinical significance of these experimental findings remains to be investigated, however. As available clinical data on tuberculosis risk under tocilizumab treatment are too limited to estimate the TB risk for this compound, screening for TB according to local practice before initiating tocilizumab therapy is recommended [59].

In addition to the risk of TB reactivation, biological therapy is also believed to increase the risk of **nontuberculous or atypical mycobacterial infections**, including *M. avium complex*, *M. chelonae*, *M. marinum* and *M. abscessus*. Nontuberculous mycobacteria are ubiquitously found in water and soil and known to cause lung infections in patients with underlying lung disease, skin and soft tissue infections and disseminated disease in severely immunocompromised patients [73]. Published data on atypical mycobacterial infections under biological therapy are scarce, with the FDA surveillance system reporting an incidence lower than TB under anti-TNF therapy [74,75], whereas the Emerging Infections Network of the Infectious Disease Society of America suggested a higher incidence than that of TB in patients receiving TNF inhibitors. A possible explanation for this difference may be that TB incidence is declining due to screening and prophylaxis for TB, which has no effect on atypical mycobacteriosis [73]. Most cases of nontuberculous mycobacteriosis were observed in patients treated with infliximab and more than half of the cases presented with pulmonary disease [73]. 

Tubach *et al*. reported a series of pneumonia cases by infection with the intracellular bacteria **Legionella pneumophila** in patients treated with TNF inhibitors. 5 out of 11 cases developed acute respiratory distress syndrome, but all recovered with appropriate antibiotic therapy. The relative risk of legionella infection in RA patients treated with anti-TNF compounds was calculated to be between 16.5 to 21.0 in comparison with the overall risk in France [76].

Cases of infection with the gram-positive intracellular pathogen **Listeria monocytogenes** have been reported for all three first generation TNF antagonists [77-79]. Listeriosis in patients treated with TNF inhibitors can present as septic arthritis [77,80,81], meningitis [82,83] or sepsis [79,84]. Slifman *et al*. report 15 cases of Listeria infection associated with anti-TNF treatment in the FDA postmarketing surveillance system, 6 of them fatal, mainly in association with infliximab treatment (14/15 cases). They estimated the US annual incidence of Listeria infection to be 43 per million in anti-TNF treated patients, *versus* 13 per million in the general population aged over 60 [85]. Experimental evidence indicates that TNF signaling plays a central role in the complex host resistance to listeria infection [86,87]. To date there are no reports linking the newer biologicals golimumab, certolizumab or abatacept with listeria infection. A single case reports describes listeriosis and hepatitis B reactivation in a leukemia patient treated with chemotherapy and rituximab [88]. 

In view of the serious course of listeria infections in immunocompromised patients, Slifman recommends physicians to advise patients receiving immunosuppressant therapy, including anti-TNF compounds, to avoid or adequately heat foods that are potential sources of *L. monocytogenes* [85]. **Visceral leishmaniasis** represents a rare complication of biological treatments, which should be suspected in patients with fluctuant fever, pancytopenia and splenomegaly, especially if coming from endemic areas. 

### Salmonella Infection

A number of case reports indicate that treatment with TNF inhibitors may lead to an increased susceptibility for infection with different salmonella species [89-91]. A Spanish cohort study found the risk of non-typhi salmonellosis in RA patients treated with biologicals at 0.73/1000 patient-years not significantly increased in comparison with either RA patients not treated with biologicals or controls from the same region without RA. However, the fact that 9/17 reported cases of salmonella infection in patients under biological therapy had severe systemic infection, suggests that biological therapy may predispose RA patients to a more serious course of disease in case of Salmonella infection [92]. 

### Viral Infections

The immunosuppressive effects of biological therapies have also been associated with increased risk for reactivation of chronic viral infections, such as hepatitis B and C, herpes zoster and even PML. 

TNF-α plays an important role in the host antiviral response, so anti-TNF treatments may theoretically increase the reactivation risk of chronic viral infections. Polymorphisms in the TNF-α promoter, leading to inadequate TNF secretion, have been shown to adversely influence the outcome of hepatitis B infection [93]. Moreover, imbalance between TNF-α and IFN-γ impairs viral clearance and promotes evolution towards chronic infection [93,94]. A recent meta-analysis reported no such association of TNF gene polymorphisms and the susceptibility to hepatitis C infection [95], although TNF production was shown to be activated in hepatitis C infection [96].

In spite of the intrinsic underlying risk of hepatitis reactivation, biological agents represent an attractive therapeutic answer to the therapeutic challenges posed by RA patients with concurrent hepatitis, in view of the well-known hepatotoxic side effects of a number of conventional DMARDs, such as MTX and leflunomide.

A number of case reports alerted clinicians to the potential danger of reactivation of **hepatitis B** under anti-TNF therapy, with sometimes serious consequences, like death or liver transplantation [97-99]. Available data on reactivation of hepatitis B under anti-TNF therapy mainly come from case reports and retrospective studies with a limited number of patients [99]. Chung *et al*. reported hepatitis B reactivation in 1 out of 8 HBsAg carriers with normal liver function and undetectable viral load [100]. Roux *et al*. found no increase in viral load in 3 patients with chronic antiHBc positive hepatitis B concurrently treated with anti-TNF and lamivudine [101]. None of the three patients with hepatitis B (treated with etanercept or adalimumab without antiviral prophylaxis) in the case series of Li *et al*. experienced rises in serum transaminases or hepatitis B viral load [102]. Kaur *et al*. reported no negative effects on liver histology after 4 months of adalimumab therapy in a patient with a transient rise in hepatitis viral load [103]. Hepatic side effects and reactivation of viral hepatitis have been more frequently reported for infliximab than for either adalimumab or etanercept. This may be due to the structural differences between these compounds [99]. 

Reactivation of viral hepatitis B has also been described in association with B cell depletion by rituximab treatment, mainly in an oncological setting [88,104]. 

Information on tocilizumab and hepatitis is limited to a case report describing long-term (6.5 years) tocilizumab therapy without adverse consequences in a patient who was later discovered to be a hepatitis B carrier [105]. The effect of inhibition of IL-6 signalling on the course of viral hepatitis remains to be elucidated, since IL-6 has been implicated in both hepatitis B related hepatocellular injury, as well as in hepatitis B viral clearance [105]. 

The risk of hepatitis reactivation of the newer TNF inhibitors, golimumab and certolizumab pegol, are still unknown, as is the hepatitis B risk under abatacept treatment.

**Hepatitis C** reactivation under biological therapy has been described.  Several retrospective studies reported no hepatitis C reactivation in a series of patients treated with infliximab or etanercept [106-110]. Li reports one patient with an increased viral load after switching from etanercept to infliximab [102], whereas the study of Cansu *et al*. describes reactivation in 2 out of 4 patients [111]. In a prospective study with 31 patients, one patient experienced drastic increase in ALT, 4 showed an increase in viral load and 19 patients were still on TNF therapy with good clinical response and stable liver enzymes and viral load after 22±11 months of follow-up [112].

Marotte reported a good safety profile of 3-months of treatment with etanercept in RA patients with concomitant hepatitis C [113]. Beneficial effects of etanercept in RA patients treated for hepatitis C with ribavirin and interferon alpha have also been reported [114,115]. 

Stable liver enzymes and hepatitis viral load were reported for a treatment regimen consisting of anti-TNF therapy in combination with cyclosporine A [116,117]. Besides its well-known immunosuppressive effects, cyclosporine also inhibits replication of the hepatitis virus [118,119] and may therefore be a good choice for patients with chronic hepatitis C infection.

**Herpes zoster** is a neurocutaneous disease resulting from reactivation of the varicella zoster virus and is characterized by a painful dermatomal rash. Complications of herpes zoster include bacterial superinfection and more importantly postherpetic neuralgia, which can cause prolonged and substantial morbidity. A condition of reduced cellular immunity increases the risk of developing an herpes zoster episode. Herpes zoster is one of the more commonly occurring infectious complications reported in RCTs of biological agents for the treatment of RA [65-67,120], but this fact must be evaluated taking into account the increased incidence of herpes zoster in RA patients in comparison with the general population. Odds ratios for herpes zoster in RA patients treated with biologicals in a US health plan database population were modestly increased (OR 1.52, 95% CI 1.03-2.23), whereas combination of biologicals with corticosteroids (OR 2.51, 95% CI 2.11-3.00) or triple therapy with biologicals, steroids and conventional DMARDs (OR 1.96, 95% CI 1.02-3.80) yielded much higher herpes zoster risks [121]. A German RA registry study reported herpes zoster incidence rates of 11.1 (95% CI 7.9-15.1) per 1000 patient-years for the monoclonal anti-TNF antibodies infliximab and adalimumab, and 8.9 (95% CI, 5.6-13.3) for etanercept, in comparison with 5.6 (95% CI, 3.6-8.3) for conventional DMARDs [120]. Studies investigating the differences in herpes zoster risk among the different TNF inhibitors yield conflicting results [120,122,123]. 

In patients under biological therapy, herpes zoster may present with atypical [124] or disseminated symptomatology [125,126].

In view of their immunosuppressive effects, the use of biologicals in **HIV** positive patients remains controversial. Although the role of TNF in HIV infection is not fully elucidated yet, it appears to contribute to HIV pathogenesis rather than to its defense [127]. A number of reports indicate that TNF inhibitors can safely be used for HIV positive RA patients refractory to conventional therapies [127,128]. One of eight HAART-treated patients with stable CD4 counts in the case series of Cepeda *et al*. experienced an infectious episode under anti-TNF treatment [128]. In a case of a psoriasis HIV-patient with low CD4 counts, etanercept treatment was stopped due to severe polymicrobial infection [129]. No studies on the use of other biologicals in HIV positive RA patients are available up to now.

**Progressive multifocal leukoencephalopathy or PML** is a rare, progressive, usually fatal demyelinating brain disease, caused by reactivation of latent JC virus, a polyoma virus. Although most cases of PML occur in settings with severe immunosuppression, such as AIDS, malignancies or overly immunosuppressed transplant patients, the disease has occasionally been described in rheumatic diseases, mostly in systemic lupus erythematosus [130]. Recently, an increasing number of PML cases in association with biological therapy with antibodies targeting immune mediators have been described [131,132]. Of relevance to RA, cases of PML have been described after treatment with rituximab [130,131, 133,134] and recently also with tocilizumab [135]. 

PML is a rare complication with an infaust prognosis. Since the diagnosis of PML is difficult and the most important therapeutic measure consists of relieving the immunosuppressed state, it is important for clinicians to be aware of its existence.

## PRECAUTIONS AND SCREENING BEFORE SELECTING AND STARTING BIOLOGICAL THERAPY

### Screening and Prophylaxis for Latent Tuberculosis

In view of the risk of TB reactivation under biological therapy, it is advisable to assess a patient’s TB history and exposure. Screening for latent TB is recommended for all biological agents, except rituximab, where clinical vigilance would suffice in view of the paucity of arguments pointing towards an elevated tuberculosis risk with this drug [59]. 

Latent tuberculosis is sometimes operationally defined as the combination of absence of TB signs or symptoms in the presence of one or more risk factors for TB (TB exposure or underlying disease), together with a positive PPD (purified protein derivative) skin test [58].

However, direct diagnosis of latent tuberculosis infection is not possible. The diagnostic tests used to identify individuals latently infected with M. tuberculosis, the *in vivo* tuberculin skin test and the *ex vivo* interferon-release assays (IGRAs), are designed to identify an adaptive immune response against the bacterium, and do not directly diagnose the presence of latent mycobacteria. Furthermore, it is currently unknown what the proportion of individuals with positive TB screening tests is that truly remains infected with mycobacteria or whether and how long the adaptive immune responses responsible for a positive test persist [136]. 

The tuberculin skin test is the classic *in vivo* TB screening test in which tuberculin PPD is injected intradermally. In the presence of a TB immune response, PPD injection is followed by appearance of an induration at the injection site. The diameter of the induration considered positive depends upon the underlying risk status of the patient. In TB screening of RA patients before the start of biological therapy indurations above 5 mm are usually considered positive. In the follow-up of patients under biological therapy an increase in induration diameter by 6mm or more would be indicative of TB reactivation [137]. Tuberculin skin testing is not very reliable in immunocompromised populations. The PPD response was shown to be subdued in RA patients [138], and influenced by previous BCG vaccination [136,139]. 

The tuberculosis-specific interferon-gamma release assay (IGRA) as an alternative screening for latent TB has been adopted so eagerly by the clinical community, as to interfere with the proper investigation of its predictive value [136]. Although a number of studies report better results in RA patients with IGRA in comparison with tuberculin skin testing [140] and good agreement between results of different IGRAs [141], IGRA testing suffers from a certain percentage of indeterminate results, necessitating the combination of both screening tests [142-145]. 

Patients with a positive TB screening test should be assessed for active disease with a chest X-ray and treated with appropriate prophylactic TB therapy. Chemoprophylaxis for latent TB usually consists of isoniazid single therapy for 9 months, or alternatively, rifampicin for 4 months [58]. In regions with TB drug resistance of >10% combination drug therapy must be considered. Liver function tests should be monitored every two to four weeks during TB treatment, especially in patients concurrently taking potentially hepatotoxic medications [146]. 

A Greek retrospective study observed 11 cases of active TB among 45/613 patients fulfilling the criteria for TB chemoprophylaxis, with 3 cases occurring in a subset of 9 patients not complying with the chemoprophylaxis scheme used. However, failure of TB prophylaxis in 8/36 compliant patients indicates that the TB prophylaxis schemes used in this study (6 months of isoniazid or isoniazid in combination with rifampicin for 3 months) were inadequate [147].

In view of the evidence pointing towards a lower risk for tuberculosis reactivation with etanercept in comparison with infliximab and adalimumab [42,148] one might at this moment consider etanercept as the treatment of choice for patients with elevated TB risk (increased TB exposure due to socioeconomic factors, proven contact with a TB case, positive tuberculin skin test), in combination with adequate TB chemoprophylaxis if necessary. However, the evidence at hand presently does not allow turning this cautious consideration into a true recommendation, in line with the recently published EULAR recommendations which do not mention any preference of one drug over another, nor take infection risk into consideration in any of the 15 recommendations [149]. 

### Hepatitis B and C Screening and Antiviral Prophylaxis

A screening and prophylaxis workup for hepatitis B in RA patients has been described by Calabrese *et al*. [150]. Prior to initiation of biological therapy hepatitis B serology should be assessed by HBsAg, anti-HBs and anti-HBc tests. Negative patients should be considered for hepatitis B vaccination. Patients positive for hepatitis B core antibodies have gone through active hepatitis infection and should be monitored closely for reactivation. Addition of antiviral prophylaxis should be considered on an individual patient basis. Periodical follow-up of liver enzymes and hepatitis B viral load is advised when no prophylaxis is given. Patients with HBsAg positivity should receive prophylaxis with antiviral drugs before starting immunosuppressive therapy. Antiviral prophylaxis with lamivudine (100 mg/day) has been used with good short-term results, while its long-term use may be involved in the development of resistant HBV strains. Little information is available on alternative antiviral therapies in RA [150].

Screening for hepatitis C virus prior to biological therapy is appropriate [127]. In view of the role of TNF in hepatitis C infection and the relative safety of TNF blockers in patients with hepatitis C infection, no change of antiviral therapy is needed, provided there is adequate monitoring of liver enzymes and viral load [112].

## HIV

HIV screening prior to biological therapy is recommended in patients with risk behavior. Biological therapy should be reserved for stable HIV positive patients with adequate (>200/ml) CD4 cell counts [127].

### Vaccination

RA patients treated with biological therapy must be regarded as immunocompromised individuals and are as such at increased risk of infection and complications for some vaccine-preventable diseases. The benefits of vaccination in this population are even greater than in the general population, but vaccination coverage is surprisingly low [151,152].

Like in all immunocompromised individuals, live vaccines (measles-mumps-rubella, varicella and zoster vaccine, yellow fever, oral poliomyelitis) are contraindicated in RA patients under biological therapy. For inactivated vaccines, biological therapy may have a negative impact on the quality of the vaccine-induced immune response. Therefore, vaccination status should be checked and updated as appropriate before the start of biological therapy. 

Live vaccines need to be given 3 to 4 weeks prior to the start of therapy to ensure clearance of the vaccine virus before the immune response is impaired. The waiting period needed before administering live vaccines after biological therapy discontinuation depends on the type, dose and duration of the therapy [153]. As a rule of thumb, a period of 3 months is estimated to be sufficient for restoration of the immune response. For rituximab, B cell repletion and adequate restoration of the immune response may require a longer period of 6 to 10 months [154].

Inactivated vaccines can be safely administered during biological therapy. Although the influenza, pneumococcal and hepatitis B vaccines have been demonstrated to be safe and effective in RA patients treated with biologicals, a number of studies indicate that the quality of the vaccine-elicited immune response in these patients is lower, with either reduced seroconversion rates after vaccination – leaving a subset of patients unprotected - or reduced quantity or quality of the antibody response to the vaccine, which in turn may have a negative effect on the duration of protection [155].

## CONCLUSIONS

Clinicians considering starting biological therapy for an RA patient should be aware that biological therapy further increases the already moderately increased infection risk of the RA patient. Precautions needed before the start of biological therapy include checking and updating the patient’s vaccination status and screening for latent tuberculosis.

Current evidence includes insufficient data from comparative studies to make recommendations concerning the choice of biological from an infection risk perspective. However, the lower risk for tuberculosis reactivation reported for etanercept in comparison with infliximab and adalimumab may cautiously prompt the consideration of etanercept as the product of choice for patients with elevated TB risk. 
